# Supplementary material for: SyNDI: synchronous network data integration framework
Source: BMC Bioinformatics. 2018 Nov 6;19:403. doi: 10.1186/s12859-018-2426-5 (PMC6219086; doi:10.1186/s12859-018-2426-5)
Supplement: Supplementary file 5 — Files of the example presented in Additional file 4. This additional file is a zip package that contains all files used in Additional file 4. Detailed descriptions of each of these files are presented in a README file included in this zip package. (GZ 30411 kb) [file 12859_2018_2426_MOESM5_ESM.gz › SyNDITools/Meme2Fimo/meme_4.9.0_4.tar.gz!/meme_4.9.0/doc/ceqlogo.xml]

ceqlogo


# ceqlogo

**Usage:** `ceqlogo -i <filename> [options]`

**Examples:**

> Load all motifs within a MEME motif file and write to logo.eps in EPS format:   
> `ceqlogo -i meme.motifs -o logo.eps -f EPS`
>
> Load second motif from each of two files and shift the first one:  
> `ceqlogo -i2 meme1.motifs -s 3 -i2 meme2.motifs -o logo.eps -f EPS`
>
> Run a self-test:  
> `ceqlogo -test`

**Description:**

> The `ceqlogo` program generates one or multiple, aligned logos
> in EPS or SVG format, based on a set of nucleotide or amino acid
> frequency matrices provided in the MEME file format.
> The code of `ceqlogo` is based on the Perl code of
> weblogo
> but is written in C and supports SVG output. Note that the program strives to use
> command line options similar to those of `weblogo`.
>
> The letter stacks are calculated by the following equations found in
> Schneider and Stephens paper
> "Sequence Logos: A New Way to Display Consensus Sequences" and adapted
> from the weblogo documentation. The height of a letter is calculated as:
>
> `height(b,l) = f(b,l) * R(l)`
>
> where `f(b,l)` is the frequency of base or amino acid `b`
> at position `l`. The stack height `R(l)` is the amount
> of information present at position `l` and can be quantified as follows:
>
> `R(l) for amino acids = log(20) - (H(l) + e(n))`
>
> `R(l) for nucleic acids = 2 - (H(l) + e(n))`
>
> where `log` is taken base `2`, `H(l)`
> is the uncertainty at position `l`, and `e(n)` is the
> error correction factor for small sample sizes `n`.
> `H(l)` is computed as follows:
>
> `H(l) = - (Sum f(b,l) * log[ f(b,l) ])`
>
> where again, `log` is taken base `2`. `f(b,l)`
> is the frequency of base `b` at position `l`. The sum is
> taken over all amino acids or bases.
>
> The error correction factor `e(n)` is approximated by:
>
> `e(n) = (s-1) / (2 * ln 2 * n)`

**Input:**

- <input filename> - A file containing one or more motifs in MEME format.

**Output:**

- If no output file is specified `ceqlogo` writes to standard output.

**Options:**

Options with arguments (all lower case):

- `-i <input filename>` - Loads all motifs within the file.
- `-i<n> <input filename>` - Loads the n-th motif within the file.
- `-s <shift>` - Shift for previously loaded motif (-i).
- `-b <bar bits>` - Number of bits in bar (real # > 0).
- `-c <tic bits>` - Number of bits between tic marks.
- `-e <error bar fraction>` - Fraction of error bar to show (real # > 0).
- `-f <format>` - Format of output (`EPS,SVG`).
  Default is `EPS`.
- `-h <logo height>` - Height of output logo in cm (real # > 0).
- `-k <kind of data>` - `AA` for amino acid, `NA` for nucleic acid.
- `-o <output file>` - Output file path. Default is stdout.
- `-n <sample number>` - Number of samples for previously loaded motif (-i).
  Used to calculate error bars.
- `-t <title label>` - Label for title.
- `-w <logo width>` - Width of output logo in cm (real # > 0)
- `-x <x-axis label>` - Label for x-axis.
- `-y <y-axis label>` - Label for y-axis.
- `-p <pseudo counts>` - Pseudo counts for motifs. Default is 1.0.
- `-test [<verbosity level>]` - Runs a self-test.
  All other options are ingored then. The verbosity level [0..3] is optional.

Toggles (all upper case):

- `-B` - Toggle bar ends.
- `-E`  - Toggle error bar.
- `-O` - Toggle outlining of characters.
- `-P` - Toggle fineprint.
- `-N` - Toggle numbering of x-axis.
- `-X` - Toggle boxing of characters.
- `-Y` - Toggle y-axis.

**Known problems:**

- SVG output is only partially implemented.

---

Author: Stefan Maetschke (s.maetschke@imb.uq.edu.au)
